# Isolated pancreatic metastasis from rectal cancer: a case report and review of literature

**DOI:** 10.1186/1477-7819-8-26

**Published:** 2010-04-07

**Authors:** Chao-Wei Lee, Ren-Chin Wu, Jun-Te Hsu, Chun-Nan Yeh, Ta-Sen Yeh, Tsann-Long Hwang, Yi-Yin Jan, Miin-Fu Chen

**Affiliations:** 1 Department of Surgery, Chang Gung Memorial Hospital at Linkou, Chang Gung University College of Medicine, Taoyuan, Taiwan; 2 Department of Pathology, Chang Gung Memorial Hospital at Linkou, Chang Gung University College of Medicine, Taoyuan, Taiwan

## Abstract

Isolated pancreatic metastases from a non-pancreatic primary malignancy are very rare. Studies have shown that resection of metastases is of proven benefit in some types of tumors. We report a case of 76-year-old Taiwanese woman with rectal adenocarcinoma treated with neoadjuvant chemoradiotherapy and abdominoperineal resection 2 years ago presenting with an asymptomatic mass at the pancreatic tail on a routine follow up abdominal computed tomography scan. The patient underwent distal pancreatectomy and splenectomy under the preoperative impression of a primary pancreatic malignancy. Histological examination of the surgical specimen showed metastatic adenocarcinoma. Immunohistochemical studies confirmed the diagnosis of pancreatic metastasis from rectal adenocarcinoma. Postoperative chemotherapy in the form of oral capecitabine was given. The patient is alive and disease free 12 months after the surgery. In a patient presenting with a pancreatic mass with history of a non-pancreatic malignancy, a differential diagnosis of pancreatic metastasis should be considered. Surgical resection of a solitary pancreatic mass is justified not only to get the definitive diagnosis but also to improve the survival.

## Background

The common sites of metastasis from colorectal adenocarcinoma are the liver, lung, and regional lymph nodes [1]. Colorectal adenocarcinoma, however, rarely metastasize to the pancreas. Isolated pancreatic metastases from non-pancreatic primary tumors are very rare, accounting for approximately 2% of all pancreatic neoplasms [2]. Renal cell carcinoma is the most common primary malignancy to metastasize to the pancreas [3-5]. Studies have shown that surgical resections of hepatic or lung metastases for colorectal malignancy patients provide survival benefit [1]. However, the role of surgery for a solitary pancreatic metastasis from colorectal adenocarcinoma has not yet been defined because of the rarity of the condition. To the best of our knowledge, very few colorectal malignancy cases with pancreatic metastases are reported in the literature [3-7]. Herein, we report a case with primary rectal adenocarcinoma with metachronous pancreatic metastasis undergoing surgical resection and also conduct a substantial review of the literature relevant to pancreatic metastases from colorectal malignancy.

## Case Presentation

**Figure 1:**
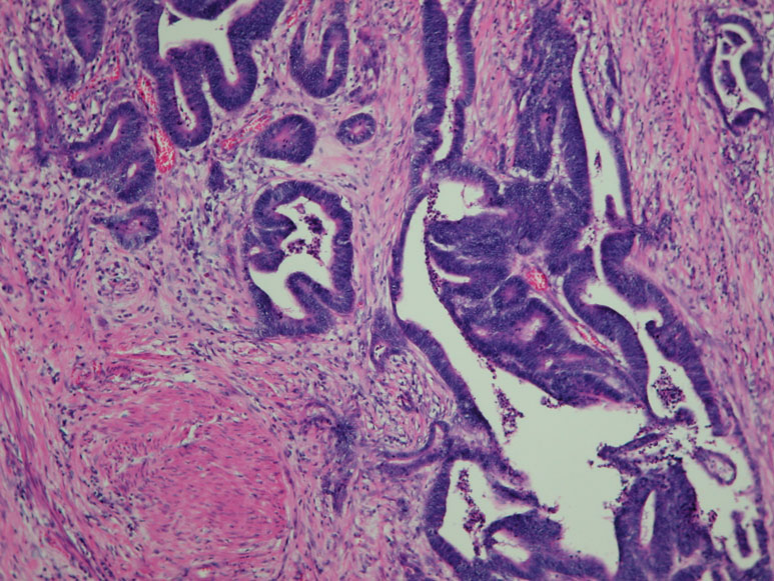
**Histological specimen of primary rectal cancer demonstrates a moderately-differentiated adenocarcinoma with invading through the muscularis propria into the subserosa (hematoxylin and eosin staining, 20×)**.

A 76-year-old Taiwanese woman had undergone neoadjuvant chemotherapy/radiotherapy and abdominoperineal resection for rectal adenocarcinoma (stage IIIa; pT3N0 M0 according to the 6th edition AJCC; Figure 1) 2 years ago. No post-operative adjuvant chemotherapy or radiotherapy was administered to the patient. She was relatively well postoperatively, without any evidence of disease recurrence or associated symptoms until she was incidentally found to have a mass in the pancreatic tail on a routine follow up abdominal computed tomography scan.

**Figure 2:**
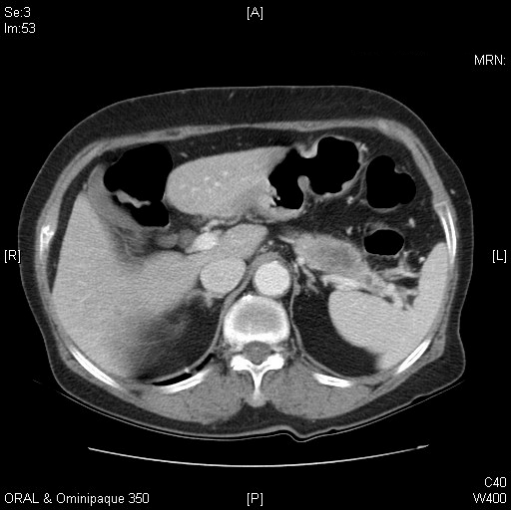
**Abdominal computed tomography reveals an ill-defined hypodense mass approximately 3.0 × 1.6 cm in diameter in the pancreatic tail**.

On admission, physical examination, hematogram and biochemistry tests were unremarkable, except for a midline operative scar and an end-colostomy. The carcinoembryonic antigen level (2.16 ng/ml) was within normal range. Abdominal computed tomography revealed an ill-defined hypodense mass measuring 3.0 × 1.6 cm in diameter at the pancreatic tail (Figure 2). There was no evidence of local recurrence of rectal cancer, lymphadenopathy or distant metastasis. A primary pancreatic malignancy was suspected, and the patient underwent distal pancreatectomy with splenectomy.

**Figure 3:**
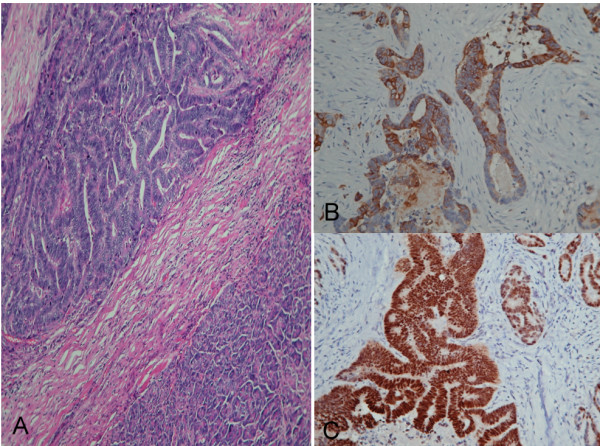
**Photomicrography of the pancreatic mass depicts a moderately differentiated adenocarcinoma with marked necrosis (hematoxylin and eosin staining, 20×; **A). Immunohistochemial stain studies of the pancreatic tumor shows positive for CK-20 (B) and CDX2 (C), further confirming the diagnosis of metastasis from rectal adenocarninoma.

Macroscopically, the cut surface of the pancreatic mass demonstrated a whitish, firm, and infiltrating tumor with ill-defined margins. Histopathological exam showed a moderately differentiated adenocarcinoma with marked necrosis (Figure 3A) which was morphologically the same as the primary rectal adenocarcinoma. Immunohistochemical studies showed the tumor cells positive for CK-20 (Figure 3B) and CDX-2 (Figure 3C), markers for colorectal adenocarcinoma, confirming the final diagnosis of pancreatic metastasis from rectal adenocarcinoma.

The postoperative course was uneventful except for fever which developed 3 weeks after surgery. Abdominal computed tomography revealed a fluid collection, 3 cm in size near the pancreatic stump suggestive of intraabdominal abscess and the patient recovered with antibiotic treatment. Post operative chemotherapy in form of oral capecitabine was given and the patient is alive and disease free 12 months after surgery.

## Conclusions

The incidence of pancreatic metastases in autopsy series performed in patients with malignant neoplasms ranged from 1.6-11% [8]. Renal cell carcinoma is the most common primary tumor, followed by lung cancer (adenocarcinoma and non-small cell lung carcinoma), lobular breast carcinoma, and more rarely, gastric cancer, melanoma, and soft-tissue sarcoma [3,6-11]. Solitary metastases to the pancreas occur even less frequently. Roland *et al *reported that 27 out of 1,357 (2%) non-pancreatic tumor patients had solitary pancreatic metastases, and resections were performed in only 4 patients [12]. Nakeeb *at al *showed that among 363 pancreatoduodenectomies (239 performed for malignant periampullary diseases), metastatic pancreatic tumors were identified in 6 cases (1.65%) [13]. Faure *et al *examined 269 pancreatic resections and found solitary pancreatic metastases in 8 cases (2.97%) [14]. In another study by Sperti *et al*, isolated pancreatic metastases were noted in 8 of 259 pancreatectomies (3%) [3]. Colorectal adenocarcinoma, however, was rarely identified to metastasize to the pancreas in those studies. Table 1 summarizes the details of colorectal adenocarcinoma cases with isolated metastasis to the pancreas in the literature and only 8 rectal adenocarcinoma cases including our patient were identified.

**Table 1:** Clinical data of colorectal cancer patients with isolated pancreatic metastases undergoing pancreatic resection in the literature

Authors	Age (years)	Sex	Site of primary tumor	Interval between primary tumor and metastases (months)	Symptoms	Site	Pancreatic surgery	Survival (months)
Roland *et al*.[12]	-	F	Colon	-	-	Tail	DP	27 ††
Nakeeb *et al*.[13]	39	M	Colon	34	No	Head	Whipple	43 ††
Harrison *et al*.[15]	-	-	Colon	15	-	Head	Whipple	41 †††
	-	-	Colon	15	-	Head	Whipple	21 †††
Inagaki *et al*.[16]	79	M	Rectum	132	No	Body-tail	DP	8 †
Le Borgne *et al*.[10]	50	M	Colon	60	Jaundice	Head	Whipple	12 †††
Tutton *et al*.[17]	37	M	Colon	23	No	Tail	DP	12 †
Torres-Villalobos *et al*.[18]	86	F	Cecum	8	Body weight loss	Body-tail-	DP	6 †
Crippa *et al*.[5]	50	M	Colon	7	No	Head	PPPD	13 †††
Matsubara *et al*.[19]	50-	M	Rectum	36	Jaundice	Head	Whipple	24 †††
Eidt *et al*.[20]	-	-	Colon	12	-	Head	PPPD	105 †††
Shimada *et al*.[21]	54	M	Rectum	44	No	Head	Whipple	8 †††
Bachmann *et al*.[22]	61	F	Rectum	24	Abdominal pain	Tail	DP	2 †
	64	F	Rectum	30	No	Body-tail	DP	10 †
Reddy *et al*.*[4]	-	-	Colon	-	-	-	-	3.2 yr**
Sperti *et al*.[7]	62	M	Colon	48	Jaundice	Head	Whipple	31 †
	71	M	Colon	0 (synchronous)	Jaundice	Head	PPPD	28 †
	59	M	Colon	10	Jaundice	Head	Whipple	17 †††
	62	F	Colon	36	Abdominal pain	Tail	DP	14 †
	41	F	Colon	24	Abdominal pain	Head	PPPD	10 †††
	76	F	Colon	0 (synchronous)	Abdominal pain	Head	PPPD	15 †††
	77	F	Colon	0 (synchronous)	No	Body	DP	5 †††
	48	M	Rectum	29	No	Tail	DP	30 ††
	57	M	Rectum	80	Jaundice	Head Tail	Enucleation DP	24 †††
Present case	76	F	Rectum	24	No	Tail	DP	12 †

-, not available; *, two cases with colon cancer; **, median cumulative survival of two cases; †, alive; ††; alive with disease;

†††, dead; DP, distal pancreatectomy; PPPD, pylorus-preserving pancreatoduodenectomy

Clinical presentations of colorectal tumor patients with isolated pancreatic metastases are quite different from that of primary pancreatic malignancy patients who frequently have abdominal pain, body weight loss, and jaundice [7,12]. As shown in table 1, only 4 patients (4/20, 20%) with pancreatic metastases presented abdominal pain and 1 had body weight loss (1/20, 5%). Six of 20 patients (30%) manifested jaundice which might be related to tumor location at the pancreatic head with mass effects [4,5,7,10,12,13,15-22]. Interestingly, 6 of 11 patients (54.5%) with tumor location at the pancreatic head did not present jaundice. However, it was remarkable that up to 45% of patients (9/20) were asymptomatic upon presentation. It was also reported that imaging studies are unable to differentiate primary pancreatic lesions from metastases by any specific manners [23,24]. These observations and findings suggested that if one had history of a non-pancreatic primary malignancy presenting a pancreatic mass with unusual manifestations during follow-up, solitary pancreatic metastasis, in addition to primary pancreatic malignancy, should be considered.

In regard of treatment of cancer patients with an isolated distant organ metastasis and the absence of widespread diseases, a number of studies have shown that resection of metastases has been proven beneficial for some types of tumors. For example, metastases to the liver, brain, and lung from tumors such as sarcoma, renal cell carcinoma, colorectal cancer, and gastrointestinal stromal tumors, metastasectomy have been reported to have salutary effects on patient survival [1,25-28]. However the role of surgery for solitary pancreatic metastases from colorectal carcinoma has not yet been well-defined. Given the fact that metastasectomies for colorectal cancer patients with hepatic and pulmonary metastases are beneficial [1,25], it seems to be reasonable to perform pancreatic resections for those patients with isolated pancreatic metastases. Table 1 demonstrated outcomes of patients after pancreatic resections for metastatic colorectal adenocarcinoma with median survival of 16.5 months. Notably, Reddy *et al *reported that a cumulative median survival of patients after pancreatic resection was more than 3 years [4]. In the current case, surgical resection is reasonable to treat and get the definite diagnosis as well as to improve patient survival. Our patient is alive with disease free more than 12 months after distal pancreatectomy and splenectomy. From a review of surgical outcomes of previously reported cases including our patient and less than 5% of surgical mortality rate in pancreatic surgery [29], we suggest that pancreatic resection for a solitary pancreatic metastasis from colorectal carcinoma is safe and feasible in a center with high volume of pancreatic surgery. The role of postoperative adjuvant therapy still remains controversial, and further studies are needed to clarify this issue.

Pancreatic metastases should be kept in mind when a patient with history of a non-pancreatic malignancy, such as colorectal adenocarcinoma presenting a pancreatic mass. Long-term follow-up with appropriate imaging studies is mandatory to detect the distant metastasis including the pancreas. Pancreatic resection for an isolated pancreatic metastasis from colorectal adenocarcinoma is feasible in selected cases. Surgical resection of a solitary pancreatic mass is justified not only to get the definitive diagnosis but also to improve the survival.

## Consent

Written informed consent was obtained from the patient for publication of this case report and any accompanying images. IRB approval was also obtained for collecting the data.

## Competing interests

The authors declare that they have no competing interests.

## Authors' contributions

LCW: data collection and analysis, drafting the manuscript. WRC: pathological review of surgical specimens, preparing histopathological figures. HJT: drafting and revising the manuscript, surgical management of the patient. YCN: revising the manuscript. YTS: revising the manuscript. HTL: revising the manuscript. JYY: revising the manuscript. All authors read and approved final manuscript.
